# Epidemiological investigations identified an outbreak of Shiga toxin-producing *Escherichia coli* serotype O26:H11 associated with pre-packed sandwiches

**DOI:** 10.1017/S0950268821001576

**Published:** 2021-08-10

**Authors:** Saira Butt, Lesley Allison, Bhavita Vishram, David R. Greig, Heather Aird, Eisin McDonald, Genna Drennan, Claire Jenkins, Lisa Byrne, Kirsty Licence, Alison Smith-Palmer

**Affiliations:** 1 National Infection Service, Public Health England, London, UK; 2 Scottish E. coli O157/STEC Reference Laboratory, Edinburgh, UK; 3 Food, Water & Environmental Microbiology Laboratory, Public Health England, York, UK; 4 Public Health Scotland, Meridian Court, 5 Cadogan street, GlasgowG2 6QQ, UK

**Keywords:** Shiga toxin-producing Escherichia coli serotype O26:H11, outbreak, whole genome sequencing, pre-packed sandwiches

## Abstract

In October 2019, public health surveillance systems in Scotland identified an increase in the number of reported infections of Shiga toxin-producing *Escherichia coli* (STEC) O26:H11 involving bloody diarrhoea. Ultimately, across the United Kingdom (UK) 32 cases of STEC O26:H11 *stx1a* were identified, with the median age of 27 years and 64% were male; six cases were hospitalised. Among food exposures there was an association with consuming pre-packed sandwiches purchased at outlets belonging to a national food chain franchise (food outlet A) [odds ratio (OR) = 183.89, *P* < 0.001]. The common ingredient identified as a component of the majority of the sandwiches sold at food outlet A was a mixed salad of Apollo and Iceberg lettuce and spinach leaves. Microbiological testing of food and environmental samples were negative for STEC O26:H11, although STEC O36:H19 was isolated from a mixed salad sample taken from premises owned by food outlet A. Contamination of fresh produce is often due to a transient event and detection of the aetiological agent in food that has a short-shelf life is challenging. Robust, statistically significant epidemiological analysis should be sufficient evidence to direct timely and targeted on-farm investigations. A shift in focus from testing the microbiological quality of the produce to investigating the processes and practices through the supply chain and sampling the farm environment is recommended.

Key Findings

- Report on the investigation of the first UK-wide foodborne outbreak of Shiga toxin-producing *Escherichia coli* (STEC) O26:H11
- Epidemiological investigations identified an association with consuming food purchased at outlets belonging to a national food chain franchise and the consumption of pre-packed sandwiches
- Highlights the challenges of detecting STEC in produce that has a short-shelf life
- Recommendation 1: Robust, statistically significant epidemiological analysis be regarded as sufficient evidence to direct timely and targeted on-farm investigations.
- Recommendation 2: Shift in focus from testing the microbiological quality of the produce to investigating the processes and practices through the supply chain and sampling the farm environment.

## Introduction

Shiga toxin-producing *Escherichia coli* (STEC) belong to a pathogenic group of zoonotic *E. coli* that cause gastrointestinal disease in humans due to their ability to produce Shiga toxin (Stx) [1]. There are two types of Stx, Stx1 and Stx2 and 10 subtypes Stx1a, Stx1c, Stx1d and Stx2a-Stx2 g [2]. Shiga toxin targets a specific subset of cells that express the receptor GB3, including podocytes, microvascular endothelial cells in the kidney, platelets, germinal centre B lymphocytes, erythrocytes and neurons [3–5]. Certain strains of STEC have the potential to cause haemolytic uraemic syndrome (HUS), a life-threatening condition characterised by renal failure, sometimes with cardiac and/or neurological complications [2–5]. The majority of STEC isolated from patients with severe symptoms also have a gene designated *eae* that encodes the protein intimin and is commonly used as a molecular marker for the locus of enterocyte effacement (LEE) pathogenicity island. Intimin is one of the large number of proteins encoded in the LEE and is required for the formation of attaching and effacing lesions on host intestinal cells [6, 7]. STEC infection damages the lining of the gut reducing its capacity to reabsorb fluids resulting in diarrhoea, which may contain blood.

The two most commonly detected STEC serotypes in the UK are STEC O157:H7 and STEC O26:H11 ([14], https://www.gov.uk/government/publications/escherichia-coli-e-coli-o157-annual-totals/shiga-toxin-producing-escherichia-coli-stec-data-2018), https://www.foodstandards.gov.scot/publications-and-research/publications/whole-genome-sequence-typing-and-analysis-of-non-o157-stec). STEC O157:H7 and STEC O26:H11 are known to colonise the gut of ruminants, such as cattle and sheep and other animals including birds, may act as transient vectors [8–10]. Transmission to humans occurs following the consumption of contaminated food or water and direct contact with animals or their environment. In household and institutional settings, secondary, person-to-person transmission of STEC has been described [11].

The outbreaks of STEC-HUS in the UK in the 1980s were caused by STEC O157:H7 and in response, laboratory methods focused on the use of cefixime-tellurite sorbitol MacConkey (CT-SMAC) agar [12]. CT-SMAC is selective for the growth of this specific serotype, thus compromising surveillance of the other STEC, including STEC O26:H11. In contrast, commercial and in-house polymerase chain reaction (PCR) assays targeting the presence of *stx* have the potential to detect STEC of all serotypes [13]. In England and Wales, surveillance for STEC O26:H11 has been continually improving since 2013, in parallel with the increasing number of frontline hospital laboratories implementing PCR ([13, 14] https://www.gov.uk/government/publications/escherichia-coli-e-coli-o157-annual-totals/shiga-toxin-producing-escherichia-coli-stec-data-2018). However, although all patients who present to primary healthcare and submit a faecal specimen are tested regardless of symptom severity, only the regions of the country where the local hospital laboratory has implemented PCR have the potential to detect STEC O26:H11. In Scotland, the burden of STEC O26:H11 for patients with severe clinical outcomes is well-established as specimens from cases with bloody diarrhoea and HUS have been forwarded to the Scottish *E. coli* O157/STEC Reference Laboratory (SERL) for STEC testing since 2002 (https://www.foodstandards.gov.scot/publications-and-research/publications/whole-genome-sequence-typing-and-analysis-of-non-o157-stec).

In October 2019, routine microbiological surveillance at SERL identified an unexpected increase in the number of faecal specimens that tested positive by PCR for *stx1* but were negative for *stx2* and *rfbE* O157 (a gene encoding a lipopolysaccharide synthesis enzyme unique to *E. coli* O157), and a multi-agency incident management team (IMT) was convened. This report describes the outbreak investigation, highlights the microbiological and epidemiological challenges encountered and makes recommendations for future practice.

## Methods

### Microbiology investigations

In the UK, faecal specimens from hospitalised or community cases with symptoms of gastrointestinal disease are cultured in local hospital microbiology laboratories for identification of *Salmonella*, *Campylobacter*, *Shigella* spp. and STEC O157:H7. In England, a sub-set of laboratories, approximately 20%, use commercial PCR assays for the detection of gastrointestinal pathogens, including STEC other than serotype O157:H7 (non-O157 STEC) ([14], Public Health England (PHE) in-house data). Faecal specimens from patients where there is a clinical suspicion of HUS and/or those testing positive for STEC by PCR and culture-negative for STEC O157:H7 on cefixime-tellurite sorbitol MacConkey agar are submitted to the Gastrointestinal Bacteria Reference Unit at PHE for confirmation by PCR and culture [15, 16]. In Scotland, all faecal specimens from patients with severe gastrointestinal symptoms, specifically bloody diarrhoea and HUS, are submitted to the SERL for PCR and culture. During the outbreak, faecal specimens testing positive for *stx1* but negative for *rfbE* O157 at both SERL and PHE were tested using a PCR for the detection of *rfbE* O26 (http://old.iss.it/binary/vtec/cont/EU_RL_VTEC_Method_02_Rev_0.pdf), while whole genome sequencing (WGS) results were pending. All strains of STEC isolated from faecal specimens were sequenced, and serotype, *stx* subtype profile and single nucleotide polymorphism (SNP) type were derived from the genome, as described previously [17–20].

## Data availability statement

FASTQ reads from all sequences in this study can be found at the PHE Pathogens BioProject at the National Center for Biotechnology Information (accession number: PRJNA315192).

### Case definitions

Confirmed: A case of STEC O26:H11 *stx1/eae* with a sequence that falls within five SNPs of the outbreak SNP type.

Previous analysis of the relatedness of isolates of STEC has shown that isolates from cases epidemiologically linked to the same outbreak fall within the same five SNP single linkage cluster [21, 22].

### Epidemiological investigations

Prospective and retrospective case ascertainment was undertaken by reviewing *stx* profiles from faecal specimens where STEC had been isolated but that were pending WGS, and by reviewing all WGS data held in the PHE and SERL databases.

Public Health Agencies in England, Scotland and Wales operate national enhanced surveillance systems for STEC [11]. Every laboratory confirmed case of STEC O157:H7 is asked to provide a detailed account of their food history, contact with animals and environmental exposures for the 7 days prior to onset of illness using a standardised enhanced surveillance questionnaire (ESQ). In England and Wales, public health follow-up of cases of non-O157 STEC focuses on those that are infected with STEC that are positive by PCR for *stx2*, because of the association between *stx2a* and severe clinical outcomes. Cases that are infected with STEC that are positive for *stx1* only strains are not routinely administered an ESQ (https://www.gov.uk/government/publications/shiga-toxin-producing-escherichia-coli-public-health-management). In Scotland, ESQs are administered to all SERL confirmed cases of non-O157 STEC regardless of the *stx* profile. Following an initial review of the ESQ data, outbreak cases were re-interviewed using a trawling questionnaire to collect a more detailed food history.

The UK posted the outbreak on the European Communicable Disease Centre's Epidemic Intelligence Information System sharing the WGS accession numbers to ascertain whether related cases had been seen elsewhere.

### Case−case analysis of exposures

Data were extracted from the national enhanced surveillance database maintained by Health Protection Scotland (HPS) for the confirmed outbreak cases residing in Scotland, and a bespoke dataset was created by combining standardised data collected from ESQs and the trawling questionnaires. The null hypothesis for testing was that there was no association between any exposure to food from any specific food outlet and being infected with the outbreak strain.

Controls were selected from among sporadic STEC cases reported in 2018 in the HPS national database. Controls were excluded if travel outside of the UK was reported in the 14 days before onset, if an ESQ was not completed, or if they were younger or older than the age range of the cases associated with the outbreak (between 15 and 64 years). Food exposures were re-coded as binary responses. Binary variables were created for each food outlet, coded as 1 if premise was mentioned as the source of any food items. Odds ratios (ORs) were calculated for each food outlet exposure comparing the odds of outbreak cases reporting the exposure with the odds of controls cases reporting the same exposure. *P* values were adjusted for multiple testing.

Multivariable logistic regressions were conducted for each exposure, adjusting for age and sex. This analysis was also repeated using all the 2018 STEC cases regardless of age and using logistic regression to adjust for age and sex.

### Case−control analysis of exposures

A further analysis was undertaken in order to include exposures reported by cases in England, and to account for overmatching in the case−case analysis by using an existing database of healthy controls. Data were extracted from the national enhanced surveillance system for the confirmed outbreak cases residing in England, and exposure data were provided by HPS for the confirmed cases residing in Scotland. The null hypothesis for testing was: There is no association between any food exposures and being infected with the outbreak strain.

Controls were selected from the national control database maintained by PHE Field Services North West, which is frequency-matched by age with all confirmed STEC cases during the same time period. Controls were excluded if travel outside of the UK was reported or if an ESQ was not completed, if gastrointestinal illness in the month prior was reported, or if they were not resident in England. Food exposures were re-coded as binary responses. ORs were calculated for each food exposure reported by at least 25% of cases, comparing the odds of outbreak cases reporting the exposure with the odds of controls cases reporting the same exposure. The Fisher's exact test was conducted to produce *P* values, to account for the small numbers of cases (<5 exposed). A multivariable logistic regression was then created to include any food item where the results of the univariate analysis showed *P* < 0.2 and OR >1.

### Food chain investigations

Food, water and environmental samples were collected by local environmental health officers (EHOs) from two food processing sites belonging to a national food chain franchise (food outlet A), and one site belonging to the food business operator (FBO) A, and transported in accordance with the Food Standards Agency Food Law Code of Practice (https://www.food.gov.uk/enforcement/codes-of-practice/food-law-code-of-practice-2015) to Food, Water and Environmental Microbiology Laboratories in Edinburgh and York in cold boxes at a temperature of between 0 and 8 °C and tested within 24 h of collection. Testing of the food samples followed PHE Standard Method F17 based on BS EN ISO 16654:2001 http://img.21food.cn/img/biaozhun/20100729/181/11294219.pdf, as previously described. STEC isolated from food samples were submitted to SERL for confirmation and typing. Growing and/or processing procedures for suppliers and wholesale distributors identified in the supply chain investigation were reviewed.

## Results

### Analysis of microbiological data

**Fig. 1.**
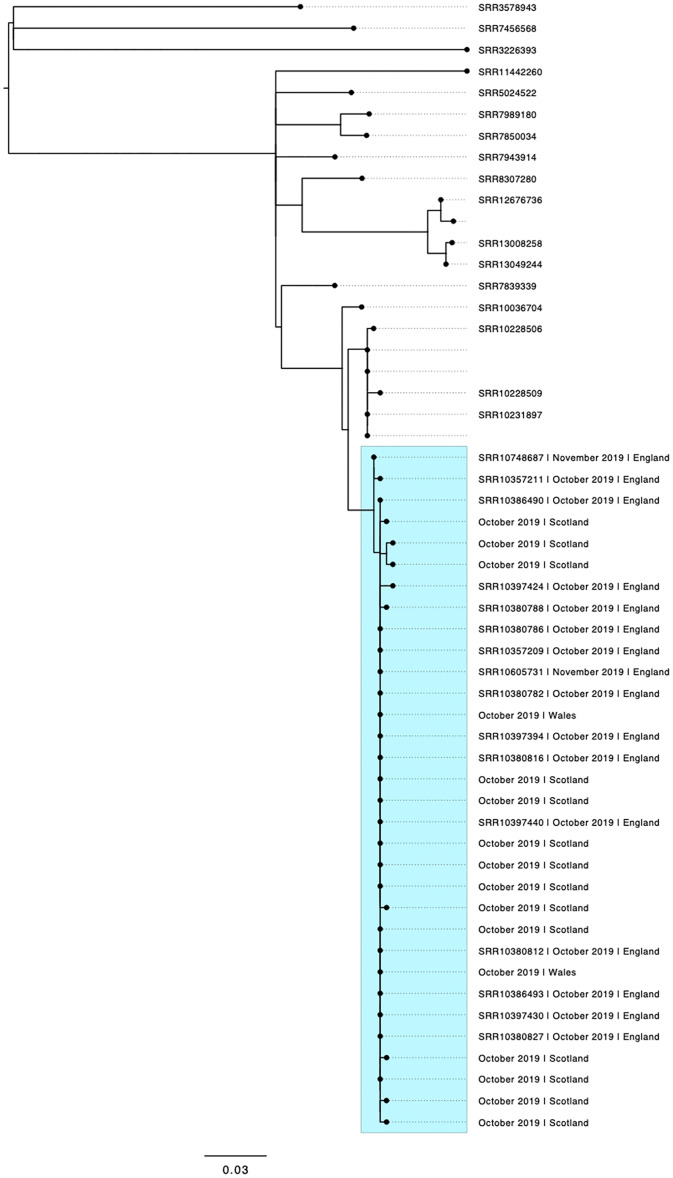
Phylogenetic tree showing the relationship between isolates linked to the outbreak from cases resident in England (*n* = 16), Scotland (*n* = 14) and Wales (*n* = 2) that fell within the same five SNP single linkage cluster highlighted in the blue box. The samples outside the blue box were included to provide context and represent all the isolates in the Public Health England (PHE) archive (2015–2019) that fell within a 25 SNP single linkage cluster of the outbreak cluster. Short read archive accession numbers (SRRs) are provided for the sequences of the isolates sequenced at PHE.

During the week commencing 14th October 2019, SERL detected a higher than average number (*n* = 11) of faecal specimens that tested positive for *stx1* compared to any other week in 2019 (median = 1.2 samples/week, range 0–6). STEC O26:H11 was subsequently cultured from 10/11 of these specimens. During the same week, PHE detected 25 faecal specimens that tested positive by PCR for *stx1* which was higher than numbers in previous weeks in 2019 (median = 10 samples/week, range 3–22). STEC O26:H11 was subsequently cultured from 11/25 of these specimens. All isolates were sequenced. Sequencing confirmed the serotype of 21 isolates (SERL = 10; PHE = 11) as STEC O26:H11, *stx1a/eae*, and all 21 isolates fell within a five SNP single linkage cluster (Fig. 1). Ultimately, between 1st October and 30th November, 32 isolates of STEC O26:H11 fell within the same five SNP single linkage cluster were identified.

### Analysis of epidemiological data

**Fig. 2.**
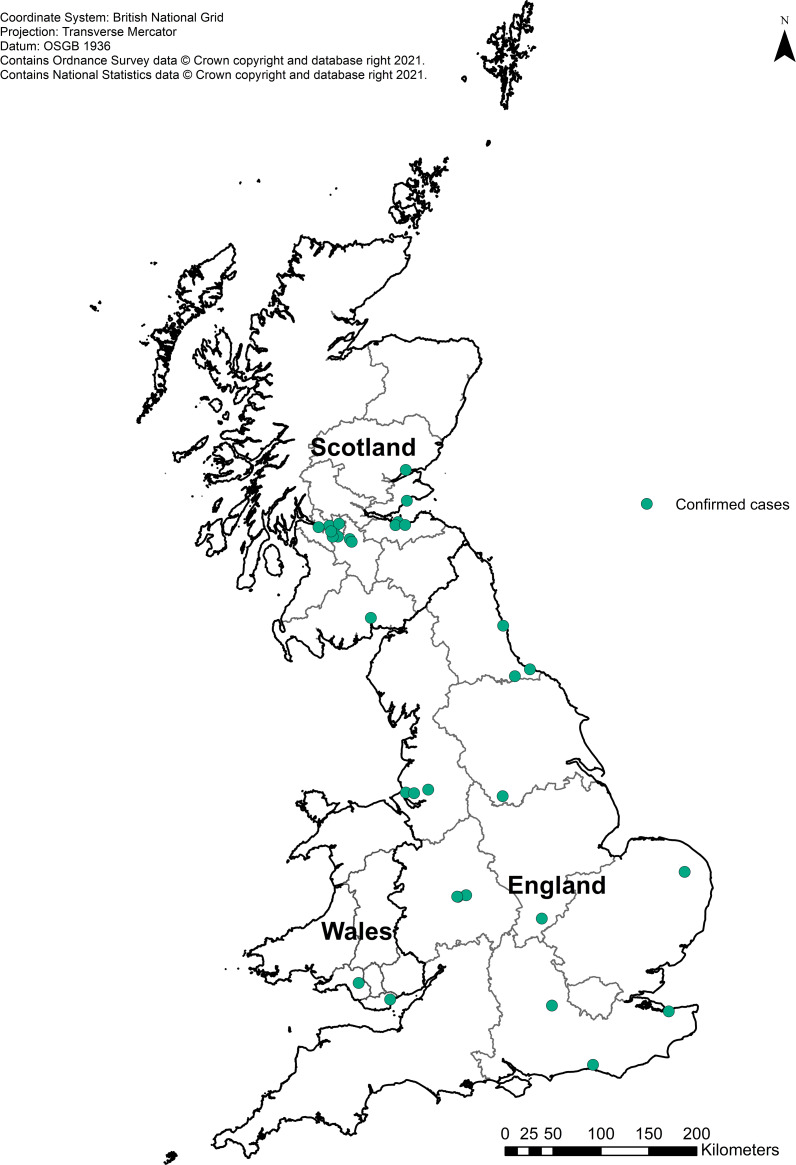
Geographical distribution of the confirmed cases (*n* = 32).

**Fig. 3.**
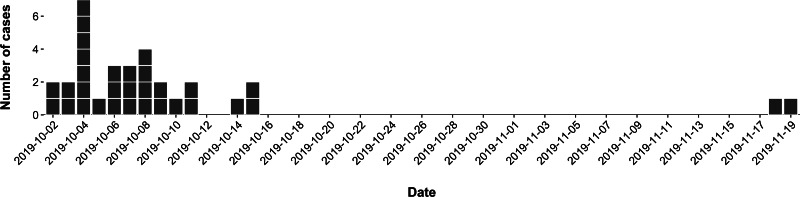
Epidemic curve of confirmed cases by onset date, or sample date where onset date was not available (*n* = 32). Both cases positioned in mid-November were based on sample dates; one case reported being symptomatic since October and no information was available for the second case.

**Fig. 4.**
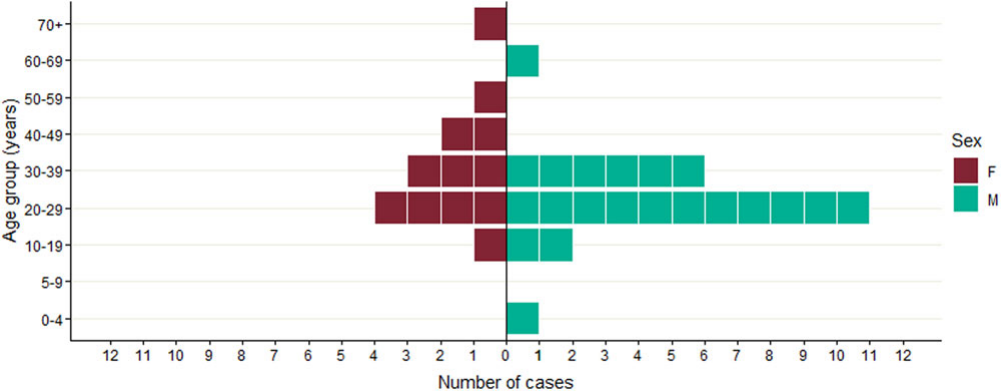
Age−sex distribution of the confirmed cases (*n* = 32).

The 32 confirmed cases were distributed across the UK, with 14 cases in Scotland, 16 in England and two in Wales (Fig. 2). Onset dates ranged from 2nd October 2019 to 18th November 2019 (Fig. 3). Twenty-one cases were male (64%) and ages ranged from 3 to 77 years of age, with a median of 27 years (Fig. 4). Clinical outcome data were available for 26 cases, of which 22 (85%) cases developed bloody diarrhoea and six (23%) were hospitalised (Table 1). No cases of HUS or deaths were reported. None of the cases reported travel abroad in the incubation period.

**Table 1.** Distribution of clinical symptoms amongst confirmed cases with a completed questionnaire

Symptoms	*n* = 26 (%)
Diarrhoea	25 (96)
Blood in stools	22 (85)
Hospitalised	6 (23)
STEC-HUS	0
Deaths	0

Exposure data were available for 28 of the 32 cases, of which 26/28 (92.9%) cases reported eating out at various food outlets within 7 days of onset of symptoms. Of these, 17/26 (65.4%) cases reported purchasing food from food outlet A, 3/26 (11.5%) cases reported purchasing sandwiches from food outlet B, and 3/26 (11.5%) cases reported eating out at food outlet C. The remaining three cases ate at three different food outlets.

### Case−case analysis of exposures

At the time of the case−case analysis, 14 cases had been confirmed in Scotland, and exposure information was available for 13 cases. In addition to the cases linked to the outbreak, a total of 221 records were extracted from the HPS database, of which 59 reported a history of overseas travel in the 14 days prior to onset and were excluded from the analysis leaving 162 cases not linked to this or any outbreak.

In univariable analysis, among food outlet exposures there was evidence of an association between being infected with the outbreak strain and consuming food purchased at food outlet A (OR = 183.89, *P* < 0.001), any bakery (OR = 17.6, *P* = 0.019) and food outlet B (OR = 13.20, *P* = 0.006) (Table 2). In multivariable logistic regressions for each food item adjusted for age and sex, association with the same exposures were maintained, with the magnitude of association increasing for food outlet A (OR = 197.05, *P* < 0.001), decreasing for any bakery (OR = 15.73, *P* = 0.030), and remaining stable for food outlet B (OR = 13.58, *P* = 0.007) (Table 3).

**Table 2.** In univariable analysis of the association between being infected with the outbreak strain and consuming food purchased at different food outlets

Variable	Estimate OR	Lower CI	Upper CI	*P* value	Composite *P* value	Adjusted *P* value	Significance
Food outlet A	183.888	23.335	2953.492	0.00000	0.00217	0.00000	1
Food outlet B	13.196	2.649	71.620	0.00053	0.00435	0.00614	1
Bakery	17.580	2.188	219.388	0.00253	0.00652	0.01942	1
Food outlet C	∞	1.295	∞	0.01608	0.00870	0.09246	0
Food Van stall/takeaway	4.176	0.908	17.901	0.04012	0.01087	0.18457	0
Indian	4.894	0.879	24.328	0.04109	0.01304	0.15750	0
Ate out	∞	0.827	∞	0.04266	0.01522	0.14018	0
Food outlet D	7.367	0.489	111.313	0.08918	0.01739	0.25640	0
Fish and chips	4.901	0.371	47.971	0.14259	0.01957	0.36441	0
Food outlet E	6.845	0.083	559.405	0.26263	0.02174	0.60404	0
Hotel	0.431	0.009	3.391	0.47631	0.02391	0.99591	0
Other restaurant/Café	1.289	0.264	5.217	0.68677	0.02609	1.00000	0
Chinese	1.309	0.208	5.918	0.69088	0.02826	1.00000	0
Food outlet F	0.000	0.000	36.178	0.75345	0.03043	1.00000	0
Nursery/School /College	0.000	0.000	36.178	0.75345	0.03261	1.00000	0
Venue Café/ Restaurant	0.715	0.015	6.054	0.83829	0.03478	1.00000	0
Italian	1.110	0.022	10.498	0.86758	0.03696	1.00000	0
Mexican	0.000	0.000	256.968	0.86869	0.03913	1.00000	0
Food outlet G	0.000	0.000	256.968	0.86869	0.04130	1.00000	0
Transport	0.000	0.000	256.968	0.86869	0.04348	0.99899	0
Pub	0.814	0.017	7.080	0.92979	0.04565	1.00000	0
Supermarket or shop	0.814	0.017	7.080	0.92979	0.04783	0.97205	0
Healthcare setting	0.941	0.019	8.481	0.97213	0.05000	0.97213	0

**Table 3.** Generalised linear model estimates based upon logistic regression adjusted for age and sex and significance level adjusted for multiple testing

Variable	Estimate OR	Lower CI	Upper CI	*P* value	Composite *P* value	Adjusted *P* value	Significance
Food outlet A	197.050	24.789	1566.374	0.00000	0.00217	0.00001	1
Food outlet B	13.584	3.061	60.274	0.00060	0.00435	0.00690	1
Bakery	15.732	2.413	102.586	0.00397	0.00652	0.03043	1
Indian	7.663	1.547	37.944	0.01260	0.00870	0.07245	0
Food van stall /takeaway	3.652	0.966	13.807	0.05622	0.01087	0.25863	0
Fish and chips	6.883	0.890	53.208	0.06450	0.01304	0.24726	0
Food outlet C	10.283	0.487	217.212	0.13429	0.01522	0.44125	0
Food outlet D	4.831	0.583	40.016	0.14422	0.01739	0.41462	0
Healthcare setting	5.483	0.287	104.751	0.25822	0.01957	0.65989	0
Hotel	0.356	0.042	3.052	0.34604	0.02174	0.79589	0
Other restaurant/ Café	1.468	0.392	5.495	0.56904	0.02391	1.00000	0
Chinese	1.355	0.320	5.729	0.67967	0.02609	1.00000	0
Venue Café/Restaurant	0.678	0.074	6.216	0.73090	0.02826	1.00000	0
Italian	1.316	0.134	12.912	0.81359	0.03043	1.00000	0
Pub	0.795	0.087	7.273	0.83922	0.03261	1.00000	0
Supermarket or shop	1.202	0.106	13.660	0.88208	0.03478	1.00000	0
Food outlet E	0.000	0.000	∞	0.98979	0.03696	1.00000	0
Ate out	0.000	0.000	∞	0.99031	0.03913	1.00000	0
Transport	0.000	0.000	∞	0.99211	0.04130	1.00000	0
Mexican	0.000	0.000	∞	0.99256	0.04348	1.00000	0
Nursery/ School/College	0.000	0.000	∞	0.99275	0.04565	1.00000	0
Food outlet F	0.000	0.000	∞	0.99283	0.04783	1.00000	0
Food outlet G	0.000	0.000	∞	0.99322	0.05000	0.99322	0

### Case−control analysis of exposures

Of 27 confirmed cases at the time of analysis (21 November 2019), questionnaires were available for 24 cases (89%), at varying levels of completion. Three hundred ninety-three controls were included in the analysis, who completed questionnaires from February to October 2019. Fifty-four per cent of controls were female, and the age range was less than 1 year to 71 years with the median age being 26 years. In univariable analysis, among food outlet exposures, there was evidence of an association between pre-packaged sandwiches and a higher OR of being a case (OR = 11.01, *P* < 0.001). The analysis also showed evidence for eating outside of the home (OR = 5.01, *P* = 0.020), handling raw beef (OR = 2.84, *P* = 0.019) and handling or consuming processed meat (OR = 2.48, *P* = 0.034) (Table 4). In the final multivariable model for food items, adjusted for adulthood and sex, nine food items were retained, of which three were associated with increased risk of the outbreak infection. As all cases were adults, controls under 15 years of age were dropped from the analysis. The most notable exposures were pre-packaged sandwiches, processed meats and raw beef (Table 5).

**Table 4.** Univariate case−control analysis of food exposures

Exposure	Cases			Controls			OR	95% confidence interval	*P* value
	Total	Exposed	%	Total	Exposed	%			
Pre-packaged sandwiches	24	17	70.83	393	71	18.07	11.01	4.12–32.37	<0.001
Other foods^1^	24	10	41.67	393	310	78.88	0.19	0.07–0.48	<0.001
Cooked poultry	24	12	50.00	393	312	79.39	0.26	0.10–0.66	0.002
Cooked beef	24	7	29.17	393	217	55.22	0.33	0.11–0.87	0.019
Ice cream	24	6	25.00	393	202	51.40	0.32	0.10–0.85	0.019
Raw beef	24	10	41.67	393	79	20.10	2.84	1.08–7.14	0.019
Ate out	24	22	91.67	393	270	68.70	5.01	1.20–44.51	0.020
Raw fruit	24	11	45.83	393	278	70.74	0.35	0.14–0.88	0.020
Fish	24	10	41.67	393	252	64.12	0.40	0.15–1.00	0.031
Processed meat	24	11	45.83	393	100	25.45	2.48	0.97–6.20	0.034
Hard cheese	24	15	62.50	393	312	79.39	0.43	0.17–1.17	0.070
Other salad	24	8	33.33	393	208	52.93	0.44	0.16–1.13	0.091
Pasteurised milk	24	16	66.67	393	317	80.66	0.48	0.19–1.35	0.114
Juice	24	7	29.17	393	181	46.06	0.48	0.17–1.26	0.139
Prepacked salad	24	6	25.00	393	124	31.55	0.72	0.23–1.96	0.651
Raw poultry	24	8	33.33	393	133	33.84	0.98	0.35–2.50	1.000
Raw vegetables	24	14	58.33	393	220	55.98	1.10	0.44–2.84	1.000
Pet/animal feed	24	8	33.33	393	128	32.57	1.04	0.37–2.65	1.000

**Table 5.** Results of multivariable logistic regression for food exposures (*n* = 225)

Exposure	OR	95% confidence interval	*P* value	Test
Pre-packaged sandwiches	60.83	4.65–795.21	*P* < 0.001	Wald
Processed meats	18.75	1.60–219.38		
Cooked poultry	7.04	0.45–110.05		
Raw vegetables	5.78	0.83–39.97		
Pasteurised milk	0.22	0.03–1.67		
Fish	0.11	0.13–0.93		
Cooked beef	0.9	0.01–1.18		
Other foods	0.07	0.01–0.58		
Soft fruit/berries	0.03	0.0–0.22		

### Food chain investigations

The ingredients list of foods consumed by the cases at food outlet A, food outlet B and food outlet C, showed the most common ingredient was a salad mix of Iceberg lettuce, Apollo lettuce and Spinach. Five samples of mixed leaf salad were taken from two sandwich preparation sites for food outlet A (Scotland = 3; North of England = 2), and one sample tested positive for a non-O157 STEC isolate. However, microbiological typing showed that the isolate belonged to STEC O36:H19 *stx2e* and was unrelated to the outbreak strain. Although this does not constitute direct evidence that this salad was the source of the outbreak as it is a different strain, it does highlight a potential pathway of human exposure to STEC from the mixed leaf salad.

The salad mix was supplied by FBO A. Test results provided by FBO A for samples of this product from 1st August to 28th October 2019, showed that samples had <10 cfu/g for *E. coli.* EHOs from the local authority for FBO A visited the food processing and distribution site, and took seven samples of the mixed leaf product supplied to food outlet A. One sample with a production date of 30/09/2019 and a use-by date of 04/10/2019 had a result for *E. coli* of 30 cfu/g but was negative for STEC. The local authority for FBO A took nine environmental swabs including swabs of drains, multi-head packers, lines and post wash product samples on the 4th November 2019. All samples tested negative for *E. coli*. FBO A reported that they routinely test environmental swabs on a weekly basis to confirm that cleaning has been effective. All swabs taken between August and October 2019 tested negative for *E. coli*. The EHOs visited the site and were satisfied with the processes that FBO A had in place.

An investigation of the growers supplying FBO A was carried out for the period between 6th of September and 22nd of October. All businesses confirmed that they did not have any retained product or soil samples from this specific time frame and that the results of microbiological sampling during the time frame under investigation had been satisfactory. During the time frame of interest, FBO A was supplied by 11 different growers, 10 farms were located in the UK and one farm was located in France. The IMT reviewed documentation, quality standards and audit reports for all growers supplying FBO A and found them to be satisfactory. Due to the large number of farms involved in the supply chain, the time that had elapsed since the production of the implicated batch of salad leaves, and the absence of an organism linking the mixed leaf salad directly to the outbreak, the IMT concluded it would be disproportionate to investigate further at the on-farm level.

## Discussion

In the UK, outbreaks caused by STEC O26:H11 occur less frequently than outbreaks of STEC O157:H7 (https://www.gov.uk/government/publications/escherichia-coli-e-coli-o157-annual-totals). It is uncertain whether this is a true reflection of differences in the burden of disease, animal reservoirs and/or transmission routes, or due to the limitations of the surveillance system for detecting cases of STEC O26:H11. Coverage of the population in Scotland is comprehensive, however, only patients infected with STEC O26:H11 presenting with bloody diarrhoea are captured, and cases with milder symptoms may be missed. Whereas in England, surveillance of STEC O26:H11 is restricted to areas where local hospital laboratories have implemented PCR. At the time of the incident, approximately 20% of local hospital microbiology diagnostic laboratories distributed across England had implemented the commercial GI PCR assay. All hospitals in Wales have implemented the PCR approach.

In England, the STEC Operational Guidance focuses on public health follow up, with respect to administering the ESQ and requiring microbiological clearance of patients in risk groups, of those cases with STEC harbouring *stx2* (https://www.gov.uk/government/publications/shiga-toxin-producing-escherichia-coli-public-health-management). Consequently, ESQs on a subset of patients linked to this outbreak and resident in England were not initially available thus hindering the epidemiological analysis in the early phase of the investigation. Evidence in the literature for the association between STEC with *stx2a* and the potential to cause HUS is well-established [2]. However, other studies have highlighted the role of *stx1a* as a marker for severe clinical outcomes, specifically as a cause of bloody diarrhoea and increasing the risk of hospitalisation [2, 23]. During this outbreak, the number of patients in England reporting bloody diarrhoea (85%) was higher when compared to those expected for STEC O157:H7, although hospitalisation rates (23%) were lower (bloody diarrhoea 61% and hospitalisation 34% from Byrne *et al*. 2015 [11]). In Scotland, hospitalisation rates for all cases of STEC in 2019 were 38% (STEC O157:H7 45%; non-O157 STEC 28%) and bloody diarrhoea was reported by 79% (STEC O157:H7 71%; non-O157 STEC 89%) https://hpspubsrepo.blob.core.windows.net/hps-website/nss/3109/documents/2_stec-in-scotland-2019-full-report.pdf. The high rates of bloody diarrhoea in non-O157 cases in Scotland may reflect the selection bias inherent in the procedure for referral of cases to SERL.

Globally, foodborne outbreaks of STEC O26:H11 have been previously associated with beef products [24, 25], dairy products [26, 27], and vegetables and salad items [28, 29]. Outbreaks of STEC O26:H11 caused by person-to-person contact, specifically in nursery school settings, have also been described [30–34]. These reports of outbreak associated with person-to-person spread provide evidence of the transmissibility of STEC O26:H11, and the need for public health follow-up of cases and exclusion of children aged five and under from school or childcare facilities, until microbiologically clear of the aetiological agent. In England, Scotland and Wales, it is also a requirement that food handlers and clinical/social care staff working with susceptible patients are microbiologically clear of the aetiological agent before returning to work.

Although the outbreak strain was not recovered from the implicated food source, the epidemiological analysis provided robust evidence that one of the components of the mixed leaf salad was the contaminated vehicle. Outbreaks caused by contaminated leafy greens and other salad vegetables are challenging to investigate because these food items are often minor ingredients of a meal that the case may fail to recall [35]. With respect to the outbreak described here, the implicated mixed leaf salad was a component of sandwich fillings, or garnish. The link between the cases was established because the majority of infected individuals reported buying takeaway food products from the same national food chain franchise (food outlet A), and other food outlets supplied by FBO A. Contamination of produce during cultivation or processing is often transient and caused by a one-off event, such as flooding, or a temporary failure in the production process caused by poor manufacturing processes [36–38]. Another problematic aspect of investigating outbreaks of STEC is that contaminated food capable of causing illness may contain the pathogen at low levels, and/or be heterogeneously distributed in the food matrix, and so detection of the pathogen is challenging, even when large analytical units of food are tested, and a robust sampling plan is in place [39, 40]. The fact that non-O157 STEC, including STEC O26:H11, are not part of the testing algorithm of food samples in the UK, and are difficult to differentiate from other non-pathogenic *E. coli* that may be present, further confounds detection.

During this outbreak investigation a strain of STEC was isolated from a sample of the mixed salad, although it was not identified as the outbreak strain. The confirmed presence of STEC in a batch of ready-to-eat (RTE) food falling is considered a serious risk to public health. Although not all strains of STEC have been found to cause bloody diarrhoea and/or STEC-HUS, a precautionary approach is appropriate given the uncertainty in the evidence and the potential for severe clinical outcomes. The FSA's current view is that the confirmed presence of STEC in RTE food is an unacceptable risk to public health and that it is appropriate to take action to remove contaminated food from the market (https://acmsf.food.gov.uk/sites/default/files/acm_1191_stec.pdf). The competent authority should be notified through incident reporting procedures and action should be taken to withdraw affected batches from the market in accordance with Article 19 of Regulation (EC) 178/2002. Information on the onward supply of the product is required to determine whether a product recall from end users/ consumers would also be appropriate. Following this incident, the product was no longer available for recall because of the time delay between the sampling and obtaining the result. However, follow up investigations were initiated by the FBO to investigate the source of STEC contamination, and the HACCP-based food safety processes were reviewed.

During the outbreak described in this study, on-farm sampling was deemed inappropriate due the lack of microbiological evidence that the mixed salad was the vehicle, the time since the contaminated product would have been on farm, the absence of evidence of ongoing contamination, and the resource commitment required. Moreover, during the time frame under investigation, the components of the mixed leaf salad had been sourced from many different farms, and that since that time there had been a seasonal shift in growers providing the supplier. However, it should be noted that STEC can survive in soil for many months [41], so even if the fields have been harvested and it has been some time since the outbreak, it may be still possible to detect the outbreak strain, and given the limitations of testing produce for STEC described above, prioritising on-farm investigations may be the most appropriate course of action. On-farm investigations during previous outbreaks linked to contaminated produce identified ruminants grazing in close proximity to the crops, and poor practice, such as the use of river or pond water for irrigation [37–39, 42]. In North America, in order to gain a better understanding of the risks associated with fresh produce there has been an increase in the emphasis placed on root cause investigations, such as sampling the farm environment, specifically soil, water sources and animal faeces, on-farm environmental audits and assessments of the processes and practices throughout the supply chain (https://www.foodsafetynews.com/2021/01/fda-tests-show-cattle-lot-implicated-in-leafy-greens-e-coli-outbreak/#more-200609, https://www.fda.gov/food/foodborne-pathogens/2020-leafy-greens-stec-action-plan).

Outbreaks of severe gastrointestinal disease caused by salad vegetables and other types of raw produce contaminated with STEC continue to be a major public health concern. Salad vegetables are sold as a ready-to-eat food, as defined in Article 14 of Regulation (EC) 2073/2005 (https://www.fsai.ie/food_businesses/micro_criteria/reg_2073_05.html). Contaminated ready-to-eat foods are regarded as a high risk compared to food that requires cooking prior to consumption. Although the production process involves a stringent washing process that removes 90–99% of external bacteria, low numbers of residual bacteria may be sufficient to cause a significant risk of infection. Outbreaks linked to contaminated produce, such as the one described here, are often transient events. Microbiological testing of food that has a short-shelf life, and where the pathogen may be present in low numbers and have a heterogenous distribution in the matrix, means that interpretation of results can be challenging as negative results do not necessarily mean the food is not the vehicle of infection. Even when PCR detects the presence of *stx* on the food matrix, confirmation of the presence of STEC can be challenging, as non-O157 STEC are not phenotypically distinguishable from other *E. coli*. Robust, statistically significant epidemiological analysis be regarded as sufficient evidence to direct timely and targeted on-farm investigations. Such an approach is essential to identify the root cause of outbreaks linked to salad and raw vegetables, and to establish an evidence-base for improving guidance and policy.
